# Supplementary material for: Evaluating large language models for diagnostic reasoning from unstructured clinical narratives in epilepsy
Source: Commun Med (Lond). 2026 May 22;6:303. doi: 10.1038/s43856-026-01653-z (PMC13197429; doi:10.1038/s43856-026-01653-z)

SemioLLM: Supplementary Materials

Supplementary Figure S1: Data preprocessing pipeline: We use Semio2Brain dataset which is a collection of 2567 semiologies spread across 7 major brain regions. Steps in preprocessing this data include removing abbreviations and replacing them with their respective full forms, correction of spelling errors present in the data, removing uninformative words and semiology categories. This result in overall 1269 rows we finally use for our analysis.

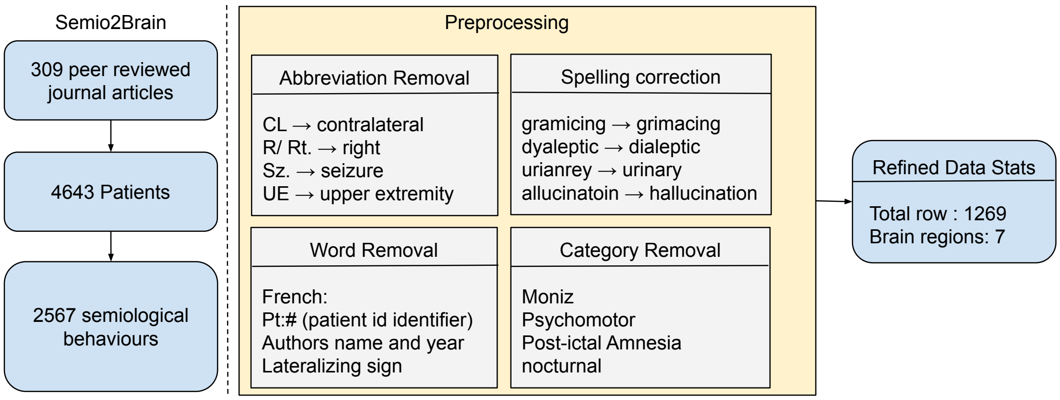

Supplementary Table S1: Performance comparison of LLMs on correctness using mean F1 scores with 95% confidence intervals. Substantial performance increase occurs with prompt-engineering [Zero-Shot (ZS), Few-Shot (FS), ZS-Chain-of-Thought (CoT), FS-CoT and Self Consistency (SC)] across all models. Mixtral-8x7B and GPT-4, which achieved substantially higher F1 scores than all other models of 51.66% and 52.27%, respectively (95% CI: [51.43, 51.90], [52.04, 52.50]), comparable to the clinician's performance: Clinician 1 - 48.77% (95% CI: [48.53, 49.02]) and Clinician 2 - 46.75% (95% CI: [46.51, 46.99]). $^{\alpha}$95% confidence intervals calculated by bootstrapping. *p $<$ 0.05 vs random performance measured using two-sided permutation test (Refer Supplementary Table S10).

LLM	F1 Scores (95% CI$^{\alpha}$) ↑				
	Zero-Shot	Few-Shot	CoT	FewShot-CoT	SC
GPT-3.5	38.17 (38.16, 38.18)	48.05 (47.83, 48.27)*	49.08 (48.85, 49.31)*	51.46 (51.22, 51.69)*	50.99 (50.75, 51.23)*
GPT-4.0	52.27 (52.04, 52.50)*	50.78 (50.54, 51.01)*	52.30 (52.07, 52.54)*	51.72 (51.49, 51.95)*	53.44 (53.21, 53.68)*
Mixtral-8x7B	51.66 (51.43, 51.90)*	45.41 (45.17, 45.65)*	52.76 (52.53, 53.00)*	52.64 (52.41, 52.87)*	50.45 (50.22, 50.68)*
Qwen-72B	39.21 (39.14, 39.29)	44.31 (44.07, 44.54)*	45.41 (45.22, 45.60)*	47.91 (47.70, 48.13)*	45.70 (45.51, 45.88)*
LlaMa2-70B	38.54 (38.47, 38.60)	39.78 (39.64, 39.91)	42.06 (41.85, 42.27)	39.92 (39.81, 40.04)	41.57 (41.37, 41.77)
LlaMa3-70B	39.84 (39.73, 39.95)*	50.06 (49.82, 50.29)*	49.21 (48.97, 49.45)*	48.03 (47.78, 48.27)*	48.65 (48.41, 48.89)*
OpenBioLLM-70B	38.56 (38.52, 38.61)	46.63 (46.38, 46.88)*	52.19 (51.96, 52.42)*	50.01 (49.78, 50.25)*	53.06 (52.82, 53.29)*
Med42-70B	45.10 (44.92, 45.28)*	31.09 (30.84, 31.35)*	46.70 (46.47, 46.93)*	45.05 (44.80, 45.30)*	47.63 (47.40, 47.87)*
Clinician 1	48.77 (48.53, 49.02)*				
Clinician 2	46.75 (46.51, 46.99)*				

Supplementary Figure S2: Clinical evaluation form illustration. (a) SOZ localization form for clinicians to provide likelihood estimates for 7 major brain regions based on 1269 semiological queries, similar to LLMs. (b) Snapshot of reasoning assessment form, where clinicians evaluate the correctness and completeness of reasoning provided by LLMs for a subset of 58 semiological queries.

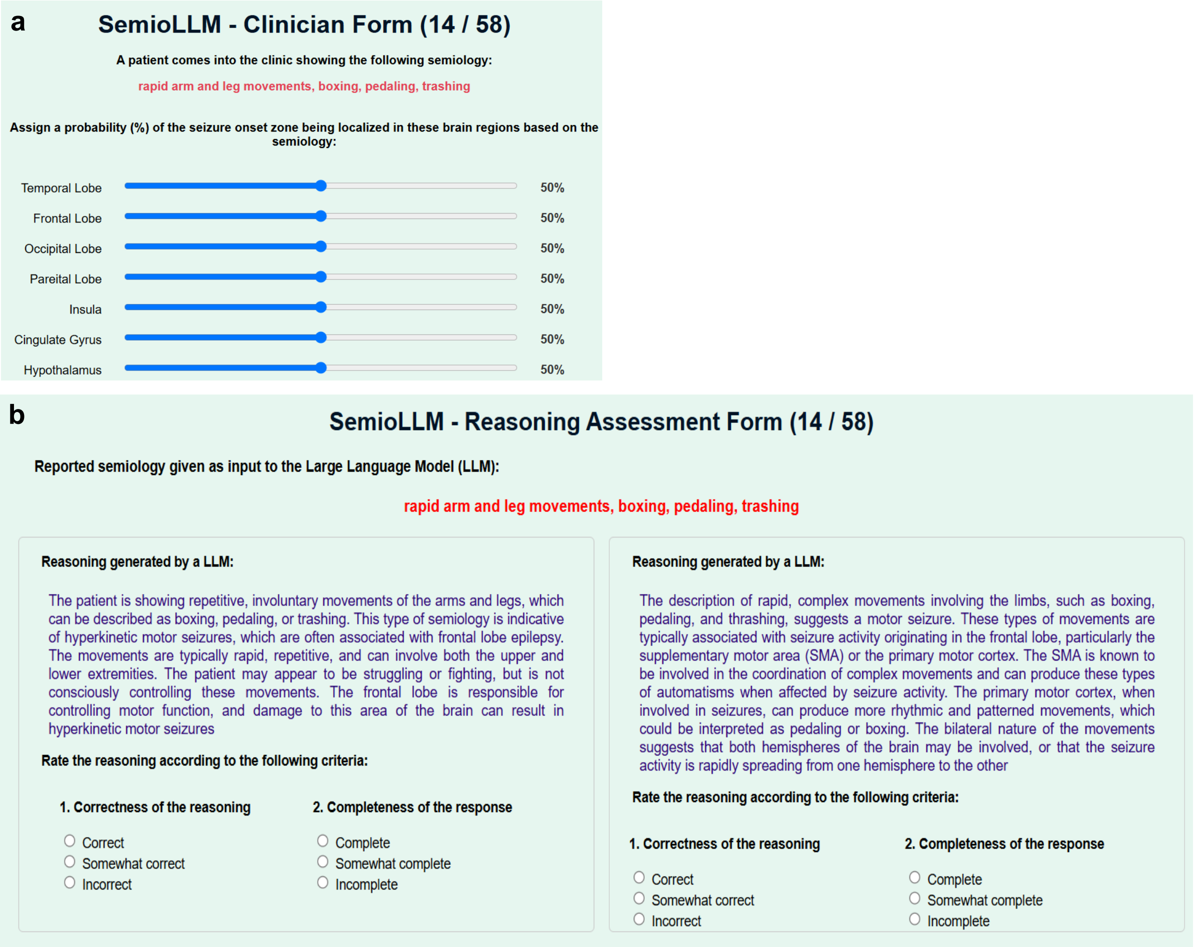
a

SemioLLM - Clinician Form (14 / 58)

A patient comes into the clinic showing the following semiology:

rapid arm and leg movements, boxing, pedaling, trashing

Assign a probability (%) of the seizure onset zone being localized in these brain regions based on the semiology:

Temporal Lobe 50%
Frontal Lobe 50%
Occipital Lobe 50%
Pareital Lobe 50%
Insula 50%
Cingulate Gyrus 50%
Hypothalamus 50%

b

SemioLLM - Reasoning Assessment Form (14 / 58)

Reported semiology given as input to the Large Language Model (LLM):

rapid arm and leg movements, boxing, pedaling, trashing

Reasoning generated by a LLM:

The patient is showing repetitive, involuntary movements of the arms and legs, which can be described as boxing, pedaling, or trashing. This type of semiology is indicative of hyperkinetic motor seizures, which are often associated with frontal lobe epilepsy. The movements are typically rapid, repetitive, and can involve both the upper and lower extremities. The patient may appear to be struggling or fighting, but is not consciously controlling these movements. The frontal lobe is responsible for controlling motor function, and damage to this area of the brain can result in hyperkinetic motor seizures

Rate the reasoning according to the following criteria:

1. Correctness of the reasoning
   - Correct
   - Somewhat correct
   - Incorrect

2. Completeness of the response
   - Complete
   - Somewhat complete
   - Incomplete

Reasoning generated by a LLM:

The description of rapid, complex movements involving the limbs, such as boxing, pedaling, and thrashing, suggests a motor seizure. These types of movements are typically associated with seizure activity originating in the frontal lobe, particularly the supplementary motor area (SMA) or the primary motor cortex. The SMA is known to be involved in the coordination of complex movements and can produce these types of automatisms when affected by seizure activity. The primary motor cortex, when involved in seizures, can produce more rhythmic and patterned movements, which could be interpreted as pedaling or boxing. The bilateral nature of the movements suggests that both hemispheres of the brain may be involved, or that the seizure activity is rapidly spreading from one hemisphere to the other

Rate the reasoning according to the following criteria:

1. Correctness of the reasoning
   - Correct
   - Somewhat correct
   - Incorrect

2. Completeness of the response
   - Complete
   - Somewhat complete
   - Incomplete

Supplementary Figure S3: Reliability diagram or calibration curves for evaluated LLMs and prompt strategies with fraction of positives (actual probability) on the y-axis and predicted probability on the x-axis. The dashed black line denotes perfect calibration.

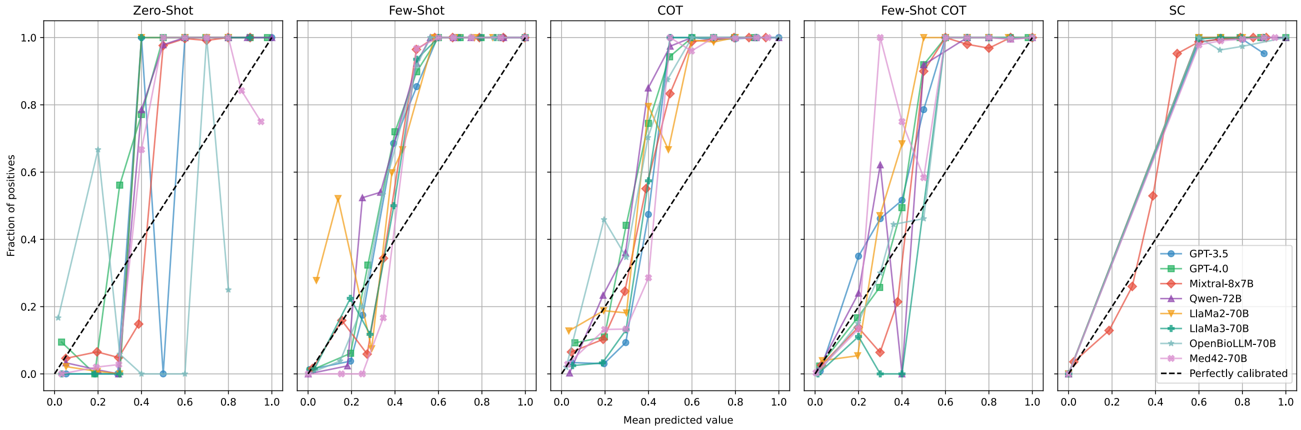

Supplementary Table S2: Performance comparison of LLMs on mean confidence score with 95% confidence intervals. Confidence improve consistently with in-context learning, with FS, FS-CoT and SC demonstrating the highest gains. 95% confidence intervals calculated by bootstrapping.

LLM	Confidence Scores (95% CI$^{\alpha}$) $\uparrow$				
	Zero-Shot	Few-Shot	CoT	FewShot-CoT	SC
GPT-3.5	0.62 (0.621, 0.621)	0.75 (0.756, 0.758)	0.65 (0.652, 0.654)	0.81 (0.818, 0.820)	0.99 (0.991, 0.992)
GPT-4	0.44 (0.439, 0.442)	0.68 (0.679, 0.682)	0.46 (0.465, 0.467)	0.68 (0.685, 0.687)	0.99 (0.999, 0.999)
Mixtral-8x7B	0.50 (0.507, 0.509)	0.63 (0.633, 0.636)	0.45 (0.450, 0.452)	0.69 (0.693, 0.696)	0.77 (0.772, 0.775)
Qwen-72B	0.60 (0.602, 0.604)	0.79 (0.796, 0.798)	0.67 (0.673, 0.675)	0.87 (0.872, 0.874)	0.99 (0.997, 0.997)
LlaMa2-70B	0.51 (0.508, 0.512)	0.70 (0.702, 0.705)	0.46 (0.463, 0.465)	0.91 (0.912, 0.914)	0.99 (0.998, 0.998)
LlaMa3-70B	0.93 (0.932, 0.934)	0.63 (0.631, 0.633)	0.60 (0.601, 0.603)	0.80 (0.806, 0.807)	0.99 (0.998, 0.998)
OpenBioLLM-70B	0.56 (0.565, 0.565)	0.86 (0.864, 0.871)	0.72 (0.721, 0.731)	0.88 (0.885, 0.890)	0.98 (0.987, 0.989)
Med42-70B	0.70 (0.703, 0.710)	0.92 (0.924, 0.929)	0.75 (0.753, 0.762)	0.90 (0.903, 0.907)	0.98 (0.985, 0.988)

Supplementary Table S3: Brier score values to estimate calibration of each model and prompt style. FS, FS-CoT and SC show improvement. Lower Brier score value indicates better calibration.

Model	Zero-Shot	Few-Shot	CoT	FewShot-CoT	SC
GPT-3.5	0.046085	0.102645	0.077054	0.034923	0.048981
GPT-4.0	0.170253	0.089790	0.140619	0.077435	0.060211
Mixtral-8x7B	0.119250	0.076820	0.125987	0.074697	0.102299
Qwen-72B	0.085287	0.093954	0.078457	0.054161	0.031092
Llama2-70B	0.177185	0.252753	0.169653	0.076720	0.047842
Llama3-70B	0.060909	0.076755	0.068321	0.018193	0.047827
OpenBioLLM-70B	0.188301	0.042845	0.098407	0.018525	0.058590
Med42-70B	0.064384	0.012571	0.045567	0.016433	0.033986

Supplementary Table S4: Impact of symptom description length on model performance. We bin the symptoms into 6 bins on word count and compute the F1 score across prompt styles for both our best performing models (GPT-4 and Mixtral-8x7B).

LLM	Bin (Word count)	#Cases	Prompt Strategy (F1 Scores)				
			Zero-Shot	Few-Shot	CoT	FewShot-CoT	SC
GPT-4	1-2	257	61.19	55.65	61.57	59.77	62.45
	2-3	391	56.13	54.44	56.09	55.13	57.50
	3-4	246	50.70	46.04	51.87	47.91	49.12
	4-6	209	42.71	45.46	40.72	42.33	45.11
	6-10	113	34.23	40.23	32.20	39.86	36.26
	10-100	53	66.09	66.83	70.97	71.55	70.97
Mixtral-8x7B	1-2	257	60.02	48.22	61.00	59.70	57.28
	2-3	391	51.11	50.05	56.53	52.26	54.74
	3-4	246	48.06	40.67	48.54	48.64	46.52
	4-6	209	47.82	36.70	44.97	49.88	41.48
	6-10	113	41.15	40.79	42.33	41.23	44.75
	10-100	53	64.64	66.21	61.68	70.68	56.28

Supplementary Table S5: Influence of in-context impersonation on zero-shot performance i.e. mean F1 scores (95% confidence intervals). We observe an increasing trend in correctness of seizure onset zone diagnosis from general persona (AI assistant) to domain aligned persona (epilepsy expert).

Model	F1 Scores (95% CI$^{\alpha}$)			
	AI Assistant	Medical Assistant	Neuroscientist	Epilepsy Expert
GPT-4	38.42 (38.39, 38.45)	38.25 (38.25, 38.25)	39.48 (39.39, 39.56)	52.10 (51.86, 52.33)
Mixtral8x7B	47.13 (46.92, 47.35)	46.53 (46.32, 46.74)	48.99 (48.79, 49.18)	51.60 (51.37, 51.83)

Supplementary Table S6: Influence of in-context impersonation on zero-shot performance confidence scores computed (1 - normalized shannon entropy). We observe and increase in confidence (or less uncertainty) trend from AI assistant to domain aligned persona (epilepsy expert).

Model	Confidence Scores ↑			
	AI Assistant	Medical Assistant	Neuroscientist	Epilepsy Expert
GPT-4	0.33	0.31	0.32	0.42
Mixtral8x7B	0.38	0.36	0.38	0.46

Supplementary Table S7: Multilingual performance analysis of GPT-4 and Mixtral-8x7B, for in-language and cross language tasks for English (En), French (Fr), Spanish (Sp) and Chinese (Ch). Notation: (X->Y) indicates input semiology in language X and model output in language Y.

Model	En→En	Fr→En	Sp→En	Ch→En	Fr→Fr	Sp→Sp	Ch→Ch
GPT-4.0	52.64	49.44	51.85	50.89	51.20	52.03	50.47
Mixtral-8x7B	53.06	52.11	50.57	49.26	47.92	48.43	38.86

Supplementary Table S8: Result of two-sided z-test for proportions for correctness and completeness in reasoning between GPT-4 and Mixtral-8x7B models. p-values<0.05 indicate statistically significant differences

Assessment Category		Z-value	p-value
Correctness	Correct	3.4893	0.0005*
	Somewhat correct	-0.6396	0.5224
	Incorrect	-3.8528	0.0001*
Completeness	Complete	3.7715	0.0002*
	Somewhat complete	-1.3939	0.1633
	Incomplete	-3.2454	0.0012*

Supplementary Table S9: Result of two-sided z-test for proportions for comparing correctness in comprehension, knowledge recall and reasoning step for FS-CoT responses from GPT-4 and Mixtral-8x7B models. p-values<0.05 indicate statistically significant differences

Assessment Category		Z-value	p-value
Correct	Correct comprehension	–	–
	Correct knowledge recall	1.1592	0.2464
	Correct reasoning step	3.8454	0.0001*
Incorrect	Incorrect comprehension	-0.7252	0.4683
	Incorrect knowledge recall	-3.5920	0.0003*
	Incorrect reasoning step	-2.4089	0.0160*

Supplementary Table S10: Permutation test results (two-sided) showing mean, standard deviation, Z-scores, and p-values (Benjamini-Hochberg corrected) for F1 scores across different models and prompt styles. p-value<0.05 shows there is a significant difference between actual model performance and random

Model	Prompt Style	Mean	Std	Z-Score	P-Value
GPT-3.5	Zero-Shot	38.291459	0.133920	-1.13438	0.283648
	Few-Shot	42.713399	1.119986	5.04679	5.89×10^{-7}
	CoT	42.263669	1.104801	6.38744	2.95×10^{-10}
	Few-Shot CoT	40.840135	1.157092	9.34291	2.62×10^{-20}
	SC	42.155762	1.099094	8.36114	1.37×10^{-16}
GPT-4	Zero-Shot	41.567860	1.072080	10.0407	7.30×10^{-23}
	Few-Shot	39.369310	1.172705	9.88929	2.23×10^{-22}
	CoT	41.345483	1.142584	9.88593	2.23×10^{-22}
	Few-Shot CoT	39.319287	1.197117	10.6888	2.41×10^{-25}
	SC	40.700700	1.163469	11.2434	1.04×10^{-27}
Mixtral-8x7B	Zero-Shot	42.701862	1.082395	8.47856	5.32×10^{-17}
	Few-Shot	38.168491	1.240102	6.00757	3.04×10^{-9}
	CoT	42.022831	1.141739	9.67015	1.54×10^{-21}
	Few-Shot CoT	41.997428	1.139507	9.62103	2.28×10^{-21}
	SC	42.235744	1.086164	7.75387	1.78×10^{-14}
Qwen-72B	Zero-Shot	38.497587	0.295359	2.49959	1.49×10^{-2}
	Few-Shot	38.450994	1.161636	5.33556	1.37×10^{-7}
	CoT	40.733622	0.872160	5.43437	8.24×10^{-8}
	Few-Shot CoT	41.179159	1.071988	6.52755	1.22×10^{-10}
	SC	40.785366	0.850382	5.84517	7.87×10^{-9}
LLaMa-v2-70B	Zero-Shot	38.541728	0.313494	-0.22767	0.860897
	Few-Shot	39.840861	0.651642	0.00206	0.983596
	CoT	42.247646	1.124966	0.06212	0.973650
	Few-Shot CoT	39.367593	0.568170	1.17384	0.2729558
	SC	42.337105	1.103209	-0.46274	0.6930558
LLaMa-v3-70B	Zero-Shot	38.644281	0.398270	3.00835	3.24×10^{-3}
	Few-Shot	38.576552	1.170794	10.0374	7.30×10^{-23}
	CoT	38.542777	1.168171	9.46883	8.48×10^{-21}
	Few-Shot CoT	36.759602	1.163803	10.0417	7.30×10^{-23}
	SC	38.261547	1.163922	9.24909	5.59×10^{-20}
OpenBioLLM-70B	Zero-Shot	38.297745	0.136530	1.95188	5.94×10^{-2}
	Few-Shot	35.278954	1.180777	9.97077	1.22×10^{-22}
	CoT	41.947639	1.135520	9.28419	4.27×10^{-20}
	Few-Shot CoT	38.798107	1.188645	9.68262	1.50×10^{-21}
	SC	40.997045	1.165039	10.6439	2.60×10^{-25}
Med42-70B	Zero-Shot	41.006967	0.827540	5.17706	3.05×10^{-7}
	Few-Shot	26.119119	1.182827	4.12808	4.65×10^{-5}
	CoT	41.091527	1.124925	5.24523	2.18×10^{-7}
	Few-Shot CoT	36.853499	1.162500	7.28592	6.09×10^{-13}
	SC	41.155962	1.101393	6.06077	2.27×10^{-9}
Clinician 1	-	37.776371	1.192979	9.47082	8.48×10^{-21}
Clinician 2	-	37.422854	1.177575	8.17473	6.22×10^{-16}

Supplementary Figure S4: Generated few-shot CoT from Llama v2 70B and Llama v3 70B model on two query semiologies; including detailed annotation of comprehension, knowledge recall and reasoning capabilities with source and SOZ likelihood. We find that LlaMa v2 does not follow the instruction to give sources, show a tendency to hallucinate patient symptoms. These issues are resolved in LlaMa v3 with better instruction following capabilities and domain knowledge.

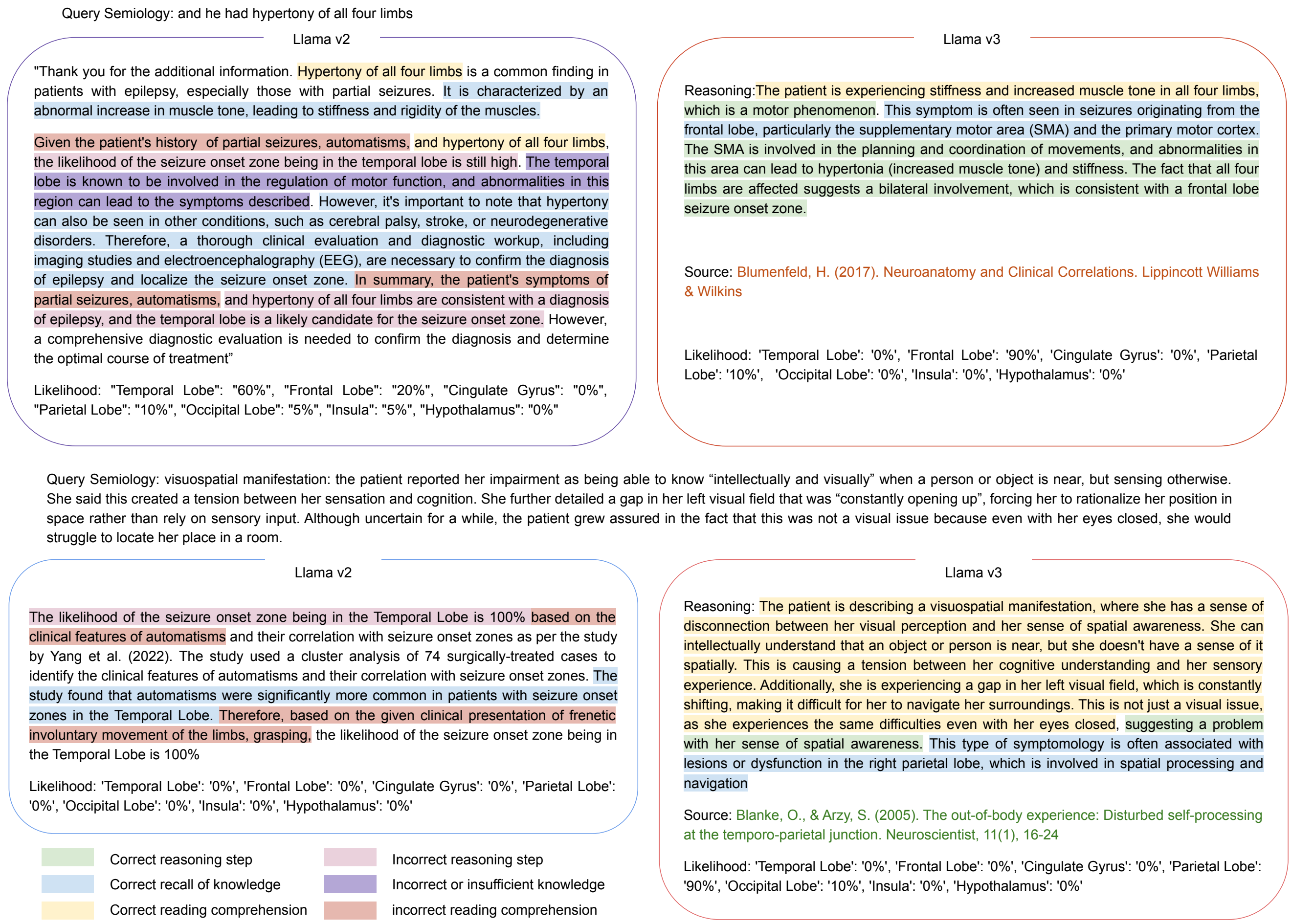

Supplementary Table S11: Statistical Comparison of LLMs and ML Models via Paired t-test. ML models use BERT and ClinicalBERT embeddings as features with Naive Bayes (NB), Support Vector Machine (SVM), K-Nearest Neighbors (KNN), and Decision Tree (DT) classifiers. * denotes p<0.05 i.e. significant difference.

	Naive Bayes		SVM		KNN		Decision Tree	
LLM	BERT	ClinicalBERT	BERT	ClinicalBERT	BERT	ClinicalBERT	BERT	ClinicalBERT
GPT-4.0 SC	2.14e-07*	3.18e-07*	8.59e-04*	1.17e-03*	5.01e-02	2.25e-01	5.83e-03*	3.19e-03*
GPT-4.0 ZS	3.54e-06*	1.25e-05*	3.02e-03*	2.00e-02*	3.16e-03	7.03e-02	7.00e-03*	2.18e-03*
GPT-3.5	4.13e-06*	1.13e-05*	7.41e-02	5.26e-01	2.42e-05*	5.17e-04*	4.96e-05*	7.13e-05*
Mixtral-8x7B	1.45e-05*	3.57e-05*	4.88e-02*	6.12e-01	1.55e-04*	1.59e-04*	1.00e-04*	4.91e-05*
Qwen-72B	4.54e-04*	2.24e-02*	1.66e-02*	9.70e-04*	1.48e-09*	4.98e-07*	1.07e-06*	9.50e-07*
LLaMA-2 70B	9.66e-03*	5.31e-01	7.21e-04*	4.01e-05*	4.51e-09*	2.97e-08*	3.14e-09*	5.62e-08*
LLaMA-3 70B	1.67e-06*	3.39e-06*	4.83e-01	3.86e-01	2.83e-05*	4.59e-04*	1.16e-05*	5.35e-05*
OpenBioLLM	2.79e-07*	3.23e-08*	2.06e-03*	5.33e-03*	5.09e-02	1.39e-01	5.56e-03*	1.72e-03*
Med42	1.45e-05*	7.14e-05*	7.52e-01	8.79e-02	2.80e-07*	4.29e-05*	5.72e-06*	6.38e-06*

Supplementary Figure S5: F1 scores for classical ML models (Naive Bayes, Support Vector Machine (SVM), K Nearest Neighbors (KNN), Decision Tree) using BERT and ClinicalBERT embeddings as features in comparison to performance of GPT-4.0 (prompt strategy: self-consistency (SC) and zero-shot (ZS)) as an end-to-end predictor on the same task and test set. Decision Tree performed comparably to the top LLMs (GPT-4.0), KNN showed no significant difference in some cases, while Naive Bayes and SVM were significantly worse than LLMs

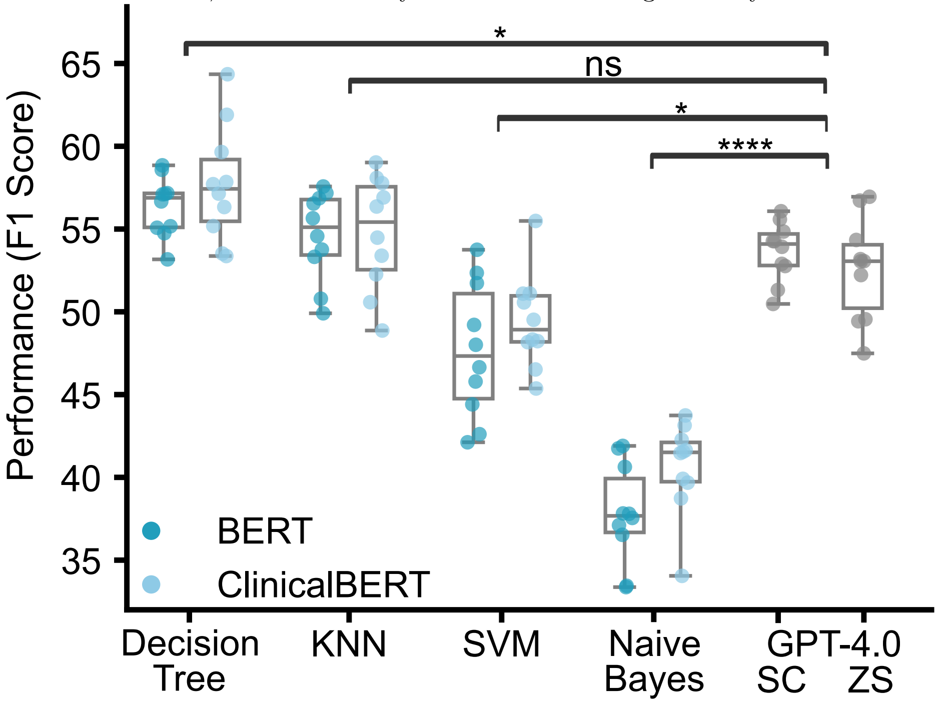

Supplement: Supplementary file 2 — Supplementary Information [file 43856_2026_1653_MOESM2_ESM.pdf]
