# Supplementary material for: Evaluating large language models for diagnostic reasoning from unstructured clinical narratives in epilepsy
Source: Commun Med (Lond). 2026 May 22;6:303. doi: 10.1038/s43856-026-01653-z (PMC13197429; doi:10.1038/s43856-026-01653-z)
Supplement: Supplementary file 3 — Description of Additional Supplementary files [file 43856_2026_1653_MOESM3_ESM.docx]

**Description of Additional Supplementary Files**

File name: Supplementary Data

Description: Source data for Figures 2(a,b), 3(b,c,d), and 4(a,b,c)
